# Smarter stomata: emergent technologies unlocking yield potential in a changing climate

**DOI:** 10.1093/aobpla/plaf048

**Published:** 2025-09-03

**Authors:** Edward Chaplin, Andrew Merchant, William Salter

**Affiliations:** School of Life and Environmental Sciences, Sydney Institute of Agriculture, The University of Sydney, Sydney, Camperdown, NSW 2050, Australia; School of Life and Environmental Sciences, Sydney Institute of Agriculture, The University of Sydney, Sydney, Camperdown, NSW 2050, Australia; School of Life and Environmental Sciences, Sydney Institute of Agriculture, The University of Sydney, Sydney, Camperdown, NSW 2050, Australia; The Australian Plant Phenomics Network, The University of Sydney, 12656 Newell Highway, Narrabri, NSW 2390, Australia

**Keywords:** stomatal phenotyping, high-throughput phenotyping, stomatal anatomy, novel plant breeding, climate resilience, wheat, abiotic stress, photosynthetic efficiency, genomic prediction, quantitative trait loci (QTL)

## Abstract

Stomata, the gatekeepers of leaf gas exchange, regulate carbon dioxide uptake and water loss, functions increasingly critical as crops face more frequent, intense heat and drought. Under dry conditions, stomatal conductance (*g*_s_) typically decreases, limiting carbon assimilation and yield. Heat stress, in contrast, elicits variable *g*_S_ responses: sometimes increasing to facilitate transpirational cooling, while at other times decreasing, especially when combined with drought. Heat and drought also induce complex, context-dependent shifts in stomatal anatomy. Smaller, denser stomata improve drought resilience in some cases, while reduced density confers greater tolerance in others. The optimal stomatal ideotype remains unknown, and different or even opposing traits may confer resilience dependent on the environmental scenario. Substantial genotypic variation in *g*_s_ and stomatal anatomy, high heritability and co-localized quantitative trait loci for stomatal traits and yield highlight their untapped potential as breeding targets for climate-resilient crops. However, stomatal traits remain largely absent from breeding pipelines due to challenges of phenotyping at scale. This is changing rapidly. Advances in deep learning, porometry, digital microscopy, and remote sensing now enable high-throughput measurement of stomatal physiology and anatomy. Next-generation breeding technologies including clustered regularly interspaced short palindromic repeats (CRISPR), multi-omics approaches, and artificial intelligence-driven ideotype selection models could revolutionize breeding, allowing precise engineering of stomatal traits for resilience to environmental stress. The time has come to move beyond characterizing stomatal traits and start actively incorporating them into breeding strategies. By leveraging these technologies, stomatal traits can become high value targets, unlocking their potential to enhance crop performance in a hotter, drier future.

## Teaser

Stomata control how plants balance carbon uptake and water loss, a function increasingly critical as crops face hotter, drier climates. Our study highlights the untapped potential of stomatal traits such as conductance, size, and density as breeding targets for improving crop resilience to heat and drought. While substantial genetic variation exists, these traits have remained underutilised due to challenges in measuring them at scale. Emerging technologies including deep learning, high-throughput porometry, digital microscopy, and remote sensing are transforming our ability to characterise stomatal traits efficiently. Combined with next-generation breeding tools like CRISPR, multi-omics, and AI-driven ideotype selection, these advances pave the way to precisely tailor stomatal traits for climate-resilient crops. Harnessing these innovations will enable breeders to integrate stomatal physiology and anatomy into breeding pipelines, unlocking their potential to improve crop performance.

## Introduction

Crucial to plant homeostasis, stomata have been a key driver in Earth’s evolutionary history (Haworth *et al.* 2021) and play a pivotal role in determining crop growth rates and yield (Pinto *et al.* 2025). Stomata are the gatekeepers of leaf gas exchange, regulating H_2_O loss and CO_2_ uptake to achieve an optimal balance between photosynthesis and transpiration. The dynamic nature of stomata are critical to optimizing crop photosynthetic efficiencies and contributing to yield potential and yield stability (Roche 2015). Stomatal traits, therefore represent high value targets for improvement in breeding programmes, however, have so far not been adopted due to difficulties in measurement at appropriate scales (Cossani and Reynolds 2012, Condon 2020).

Stomatal conductance (*g*_s_), the rate that CO_2_ or water vapour diffuse through the stomata (Wall *et al.* 2022), is determined in the short-term by dynamic physiological responses to the environment (Faralli *et al.* 2019, Li *et al.* 2022a, 2022b, Lawson and Leakey 2024). However, stomatal anatomy (guard cell size and pore density), largely fixed after leaf expansion, primarily determines *g*_s_ and the theoretical maximum potential stomatal conductance (*g*_smax_) in the long term (Franks *et al.* 2009, Dow *et al.* 2014, Mano *et al.* 2023).

**Figure 1.**
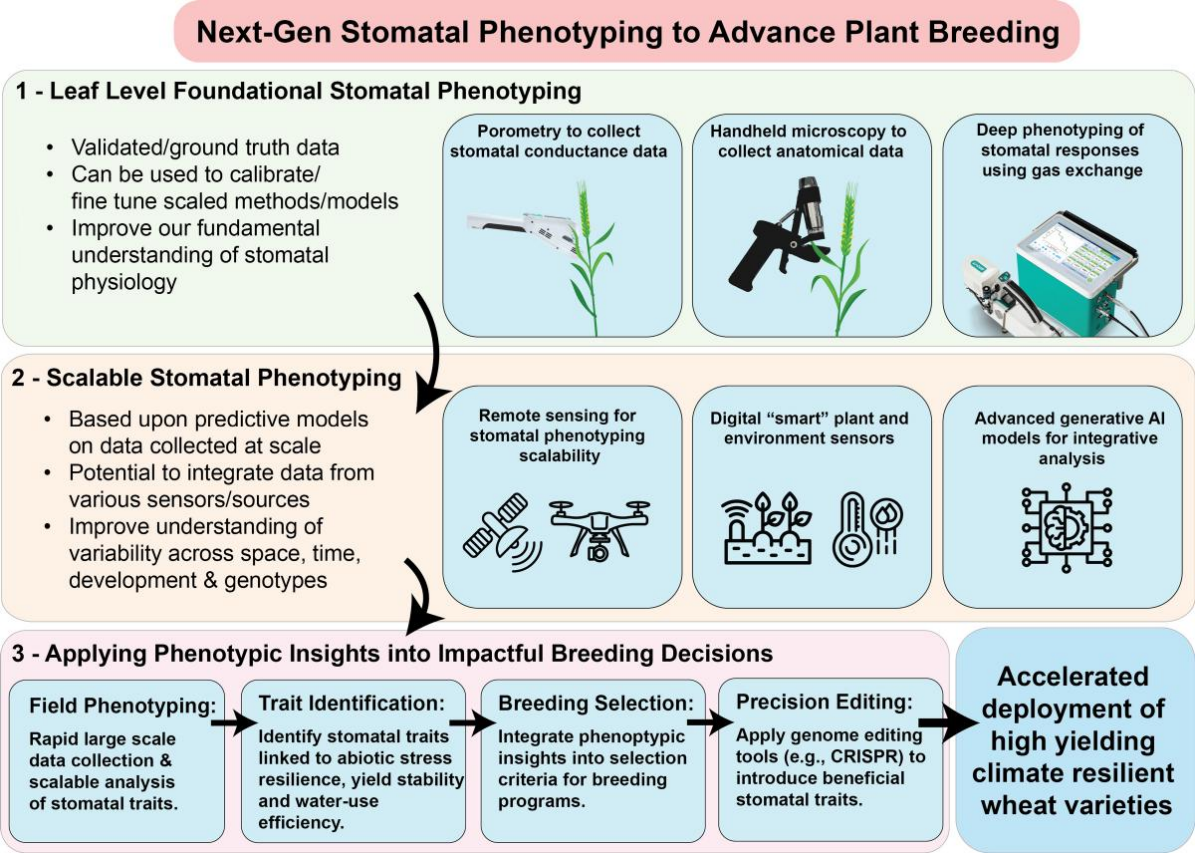
Schematic representation of advances in approaches to stomatal phenotyping, outlining (1) current phenotyping approaches, (2) future novel techniques, and (3) how phenotypic insights can lead to rapid deployment of stomatal traits in breeding programmes for impactful breeding outcomes.

Until recently, conventional phenotyping techniques limited the ability to accurately collect coupled physiological and anatomical stomatal data at breeding-relevant scales in the field. Emerging ground- and aerial-based technologies enable data collection at unprecedented field scales, providing new opportunities to optimize stomatal traits for enhanced crop performance. This article discusses the importance of new technologies and analytical approaches that will help us realize the potential of these small pores for big impacts to future crop productivity (Fig. 1). We focus on wheat due to its global importance and the need to enhance its heat and drought tolerance to maintain food security amid climate change.

### Stomatal traits as drivers of crop productivity and stress resilience

The literature presents a varied and often context-dependent picture of how *g*_s_ responds to abiotic stress. While many studies in wheat report reduced *g*_s_ under heat or drought, reflecting a water-conserving strategy (Ouyang *et al.* 2017, Balla *et al.* 2019, Ramya *et al.* 2021, Wang *et al.* 2024a), others show that *g*_s_ responses can differ markedly depending on environmental conditions, growth stage, and genotype. For instance, heat stress alone has been reported to increase *g*_s_ to enable transpirational cooling, particularly among heat-tolerant cultivars (Djanaguiraman *et al.* 2020, Abdelhakim *et al.* 2021). Combined heat and drought stress often exacerbate *g*_s_ responses to stress, beyond those observed under individual stresses (Ru *et al.* 2023, 2024). This variability underscores the complexity of environmental control over stomatal physiology and highlights the importance of considering species- and context-specific factors when interpreting the role of stomata in stress adaptation.

Significant genotypic variation exists in *g*_s_ responses to stress (Thapa *et al.* 2018, Pooja and Munjal 2019, Ramya *et al.* 2021, Wang *et al.* 2024a), with high heritability ranging from 50% to 80% (Rebetzke *et al.* 2001, 2003, Romena and Farshadfar 2019). This highlights potential for targeted selection of stomatal traits to improve productivity and resilience. While *g*_s_ itself has not been widely used directly, indirect selection via carbon isotope discrimination (δ¹³C) has identified germplasm with improved transpiration efficiency in Australian wheat breeding (Condon *et al.* 1987). Similar work in Arabidopsis identified *erecta*, a key gene regulating stomatal development (Masle *et al.* 2005). Despite their central role in determining yield, photosynthesis and related physiological traits have remained largely inaccessible to plant breeders (Schapendonk *et al.* 2007, Li *et al.* 2021). Nonetheless, yield-focussed selection has inadvertently selected lines with higher *g*_s_ (Roche 2015, Aminizadeh *et al.* 2024). Consequently, advances in phenotyping could enable direct selection for optimal stomatal traits, unlocking gains in yield and stress resilience.

Regarding stomatal anatomy, guard cell width and length tend to decrease under heat and drought, while stomatal density increases (Wang *et al.* 2016, Kapadiya *et al.* 2017, Lakde *et al.* 2024), especially in stress-tolerant varieties (Baloch *et al.* 2013, Guizani *et al.* 2023). This allows maintenance of *g*_smax_ while speeding up stomatal responses to dynamic environmental changes (Franks and Farquhar 2007, Franks and Beerling 2009, Lawson and Blatt 2014, McAusland *et al.* 2016). This trait combination can maximize water-use-efficiency (WUE) and contribute to 5%–14% yield improvement under co-occurring heat and drought stress, demonstrating that stomatal traits can translate into yield gains (Shahinnia *et al.* 2016). Guizani *et al.* (2023) and Pinto *et al.* (2025) support the notion that selecting genotypes with smaller stomata at higher density is beneficial for realizing improvements in WUE and carbon gain.

However, this trend is not universal. As outlined in Box 1, Dunn *et al*. (2019) demonstrated that reducing stomatal density through genetic manipulation can enhance WUE. Similarly, other studies report lower stomatal densities in drought-tolerant cultivars and reductions under drought stress (Li *et al.* 2017, 2023, Ding *et al.* 2018), contrasting with studies advocating for higher stomatal density. This highlights that different, and even opposite, stomatal trait combinations may confer resilience to different types of stress (e.g. long-term water deficit vs acute drought) or environmental scenarios. Increases in density may also result from reduced leaf expansion rather than direct developmental control (Doheny-Adams *et al.* 2012). The optimal stomatal ideotype likely varies with context, and at present, remains an open question requiring further research.

Box 1. Manipulating stomata to improve water use efficiency—case study.

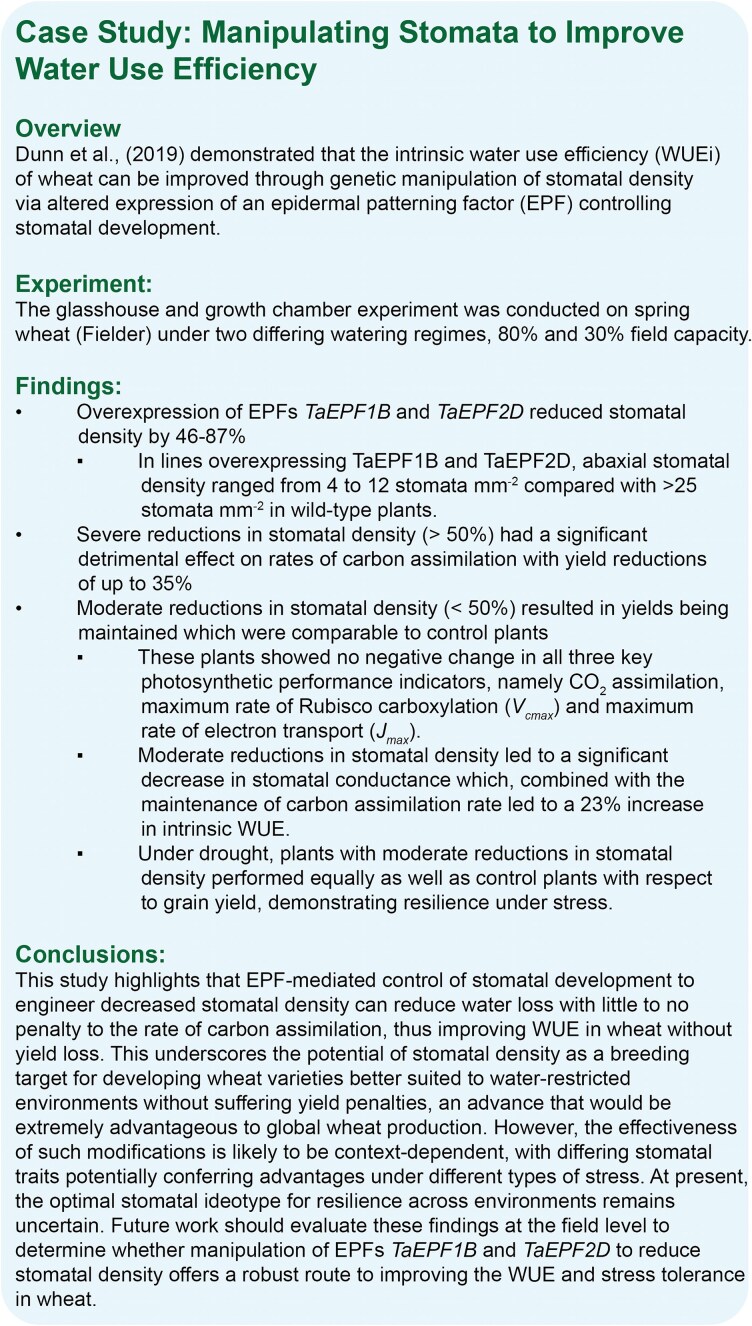

Significant genotypic variation in stomatal size and density exists in wheat, primarily driven by the adaxial surface where stomata also respond faster (Wall *et al.* 2022, Faralli *et al.* 2024, Pinto *et al.* 2025). Heritability estimates for stomatal density (68.0%), length (78.4%), width (90.2%), and area (86.1%) highlight strong genetic control (Mehri *et al.* 2008, Lamari and Fayt 2015, Romena and Farshadfar 2019). Quantitative trait loci (QTL), some pleiotropic for yield, have also been identified for stomatal traits (Shahinnia *et al.* 2016, Ahmed *et al.* 2021, Liu *et al.* 2025). However, most research on adjustments to stomatal anatomy has been under glasshouse conditions leaving stomatal anatomical dynamics in field-grown plants comparatively underexplored.

The potential of physiological traits to improve crop performance is increasingly acknowledged, but no trait operates in isolation. A holistic approach integrating physiological processes like *g*_s_ to agronomic outcomes and environmental variables is essential (Yan *et al.* 2019, Yan 2021). Focusing solely on leaf gas exchange without considering water-use or carbon assimilation could risk unintended trade-offs, such as reduced drought tolerance or inefficient resource allocation. Integrating multiple traits facilitates identification of QTLs common across traits, bridging scales and building a comprehensive framework for optimizing stomatal function (Negisho *et al.* 2022). This integration must extend to the coordinated study of stomatal physiology and anatomy, two intrinsically linked dimensions. Stomatal anatomy (pore size and density) directly influences stomatal physiology (behaviour and responsiveness), and studying both traits allows trait interactions to be unravelled (Wall *et al.* 2023, Cheng *et al.* 2024). Considering anatomically-determined *g*_smax_ alongside operating stomatal conductance (*g*_sop_) offers insights into leaf gas exchange operating efficiency, a key parameter for crop improvement (Busch *et al.* 2024, Ochoa *et al.* 2024).

Several studies show anatomical traits such as pore size and density correlate closely with *g*_s_ under abiotic stress (Baloch *et al.* 2013, Ouyang *et al.* 2017). The adaxial leaf surface mainly drives *g*_s_ changes under stress, often reflecting more pronounced anatomical adjustments than the abaxial surface (Wall *et al.* 2022, 2023, Guizani *et al.* 2023, Faralli *et al.* 2024, Pinto *et al.* 2025). Combining anatomical and physiological trait data provides deeper insight into trait combinations that optimize CO_2_ uptake and water-use. In particular, coupling stomatal density with the dynamic *g*_s_ responsiveness is key to identifying genotypes with enhanced drought resilience and water-use efficiency.

### Phenotyping stomatal traits, the here and now

Research to date has mostly been limited to a small number of genotypes and has largely focussed on individual stomatal traits as opposed to the co-occurring responses of multiple traits. While low-cost approach such as nail varnish peels and manual stomatal measurement remain effective for small-scale studies, the integration of stomatal traits as targets in breeding programmes has been constrained by the difficulty of collecting anatomical and physiological data simultaneously, and at the scale (Fig. 1). This applies to infra-red gas analysers for measuring *g*_s_ (Toro *et al.* 2019), nail polish impression techniques (Wall *et al.* 2022, 2023) and largely manual image analysis workflows (Zhang *et al.* 2025a). However, recent advancements in instrumentation and techniques, including handheld porometry with improved accuracy (McAusland *et al.* 2023), the Dynamic Assimilation Technique for faster gas-exchange measurements (Tejera-Nieves *et al.* 2024), field-portable digital microscopy (outlined in Box 2) (Pathoumthong *et al.* 2023), and deep learning computer vision approaches (Gibbs and Burgess 2024), now provide opportunities to integrate physiological and anatomical trait data at scale to realize the potential of stomata as high-value targets in plant breeding programmes. Knowledge gaps also remain in understanding dynamic stomatal responses including the speed of stomatal opening and closure due to limitations to quantification under field conditions. Stomata are the only nonspecialized plant organ capable of physically responding in an almost instantaneous timeframe to dynamic changes in environmental conditions. Understanding such responses will be crucial to defining stomatal ideotypes that optimize WUE and photosynthetic performance under varying environmental conditions (McAusland *et al.* 2016, Burgess and Ugalde 2024).

Box 2. Using deep learning to analyse stomatal anatomy—case study.

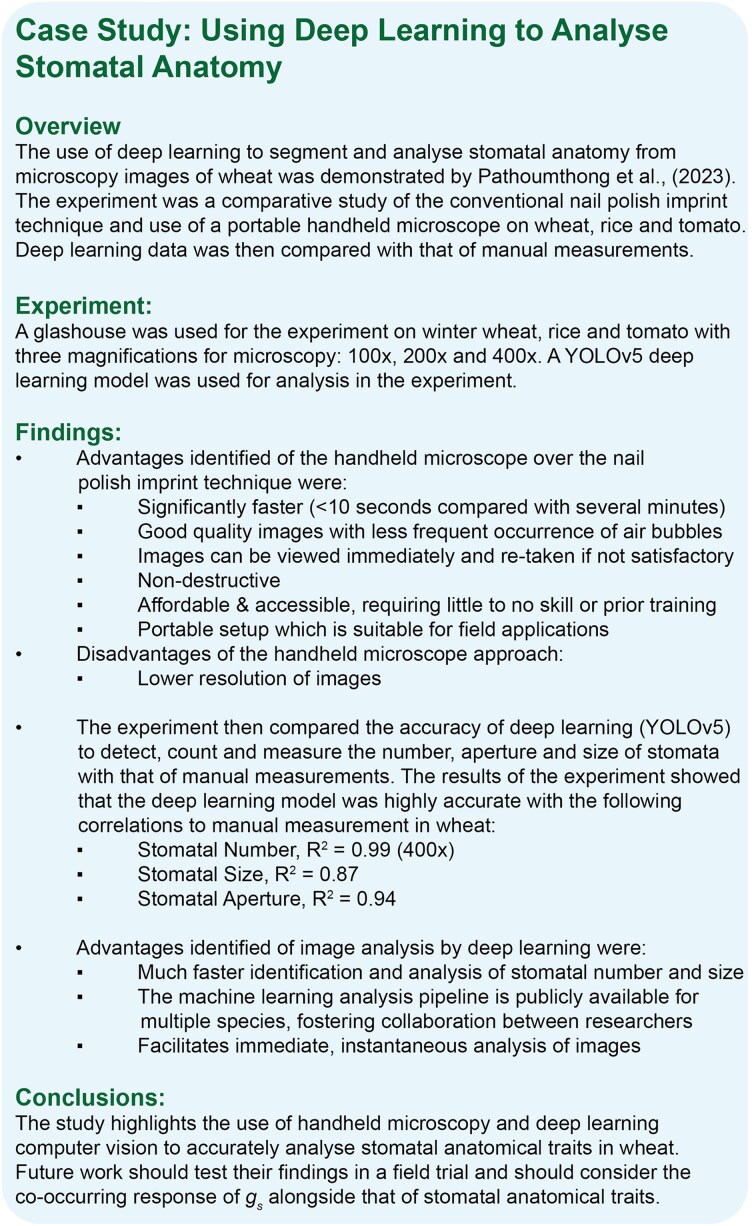

While phenotyping tools that measure stomatal behaviour, photosynthesis, and related traits at appropriate scales are crucial, they represent only one piece of the puzzle. The extended challenge lies in interpreting data from these tools and understanding their context for breeding targets under future climates. This requires a comprehensive integration of knowledge regarding stomatal dynamics, photosynthesis, plant growth, and yield through modelling to predict plant responses under dynamic environmental conditions (Buckley 2017, Vialet-Chabrand *et al.* 2017). However, these models must be fine-tuned using empirical, ground-truthed data to ensure their accuracy and applicability by combining them with higher-throughput phenotyping tools. This integration marks a pivotal step forward in plant breeding, ultimately guiding the development of crop varieties that are resilient and efficient in future climates.

### Novel approaches to phenotype stomatal traits

#### High throughput porometry and microscopy

High-throughput methodologies using new equipment like handheld porometry and handheld microscopy offer significant opportunities to advance stomatal research (Fig. 1). While conventional gas exchange measurement techniques remain valuable for understanding detailed plant responses across a small number of genotypes, accurate handheld equipment enables high-resolution anatomical and physiological data collection in the field across large germplasm populations at scales previously unattainable (Toro *et al.* 2019, McAusland *et al.* 2023). Deep learning and computer vision allows researchers to analyse thousands of images quickly, automating the segmentation and quantification of stomatal features including density, size and pore characteristics (Pathoumthong *et al.* 2023, Gibbs and Burgess 2024). While the time taken to grow plants under diverse environmental conditions remains a bottleneck in stomatal trait screening, the shift from manual to automated analysis of anatomical trait data can significantly ease downstream trait analysis. The scalability of these tools makes them particularly valuable for rapid and precise phenotyping in global breeding programmes, opening new possibilities for understanding how various stomatal anatomical characteristics influence physiological traits (Arya *et al.* 2022). Streamlining the use of field-portable porometry and microscopy equipment enables real-time integration of anatomical, physiological and environmental data, providing immediate insights into stomatal behaviour and its impact on plant performance under varying conditions (Chaplin *et al.* 2025). They also facilitate the rapid collection of reliable ground-truth data, enhancing confidence in high-throughput remote sensing approaches for stomatal assessment. However, despite the benefits of high-throughput phenotyping, the value of high-quality reliable datasets, often smaller in size, must be balanced against the desire for inherently more variable, high throughput datasets.

Recent advancements in micro-nano technology and flexible electronics have enabled the development of highly sensitive smart plant sensors that can also enhance data collection and analysis of physiological traits in the field in real-time and across time/plant development (Giraldo *et al.* 2019). These sensors can be integrated into wearable tape-based devices that continuously track factors like leaf water potential, nutrient status, and photosynthetic activity (Oren *et al.* 2017). Additionally, micro-electromechanical systems technology has enabled the creation of miniaturized sensors that attach directly to plant tissues, offering higher sensitivity, shorter response times and greater precision. While still an emerging area, this new era of wearable plant sensors has demonstrated potential for plant growth monitoring, detecting early stress responses and disease onset (Nassar *et al.* 2018, Kim *et al.* 2019, Ibrahim *et al.* 2022). Flexible hydration-status monitors (Oren *et al.* 2017, Im *et al.* 2018) and canopy temperature/humidity sensors have also been developed (Yin *et al.* 2021a, 2021b), both of which could advance stomatal phenotyping research.

Combined with advanced data analytics and artificial intelligence (AI), these cutting-edge sensors allow rapid and accurate, real-time noninvasive assessment of plant health and stress responses, paving the way for more efficient and sustainable agricultural practices (Zhang *et al.* 2025b). Despite their promise, challenges including synchronous biological sensor-plant integration (e.g. to avoid stomatal obstruction), cost, scalability and integration into existing systems remain barriers to widespread adoption in plant phenotyping (Sun *et al.* 2023, Zhang *et al.* 2023a). However, with continued development, such systems could become commercially viable within 3–5 years, opening exciting opportunities and accelerating the transition to a future of ‘smart agriculture’ (Soussi *et al.* 2024, Kuruppuarachchi *et al.* 2025).

#### Spectral prediction of physiological traits

Stomatal traits are highly dynamic making conventional sampling methods challenging and unreliable due to environmental changes during lengthy screening campaigns. The absence of high-throughput phenomics tools has long hindered breeding efforts for photosynthesis-related traits (Tao *et al.* 2022). Rapid, scalable methods are needed to capture the diurnal dynamics of stomatal traits and behaviours, enabling selection of superior germplasm from diverse populations (Furbank *et al.* 2019, Fu *et al.* 2019, Meacham-Hensold *et al.* 2019). Hyperspectral (HS) reflectance offers a novel way to rapidly characterize multi-dimensional leaf traits within relevant timeframes at unprecedented scales (Fu *et al.* 2020, Liu *et al.* 2023), with spectroradiometers capturing spectral signatures efficiently at the leaf scale (Silva-Perez *et al.* 2018, Sherstneva *et al.* 2024). Machine learning models, trained on ground-truthed physiological data, translate spectral data into accurate trait prediction, as demonstrated in wheat for Rubisco carboxylation capacity (*V*_cmax_) and electron transport rate (Silva-Perez *et al.* 2018), leaf dark respiration (*R*_dark_) (Coast *et al.* 2019) and net photosynthetic CO_2_ assimilation (*A*) (Furbank *et al.* 2021). As outlined in Box 3, promising gains have also been made to reliably predict *g*_s_ and stomatal anatomical traits using spectral data across field trials in wheat (El-Hendawy *et al.* 2019, Cheng *et al.* 2024), and in maize, cotton and other crop species (Jarolmasjed *et al.* 2018, Vitrack-Tamam *et al.* 2020, Mohd Asaari *et al.* 2022). While demonstrating indirect stomatal phenotyping at scale, further research and development is needed to refine these models and integrate them with other high-throughput phenotyping tools. The biggest barrier, however, remains collecting robust physiological data to train machine learning models. Addressing these issues could make HS phenotyping a cornerstone of stomatal trait analysis.

Box 3. Using leaf spectral data to predict stomatal anatomical traits—case study.

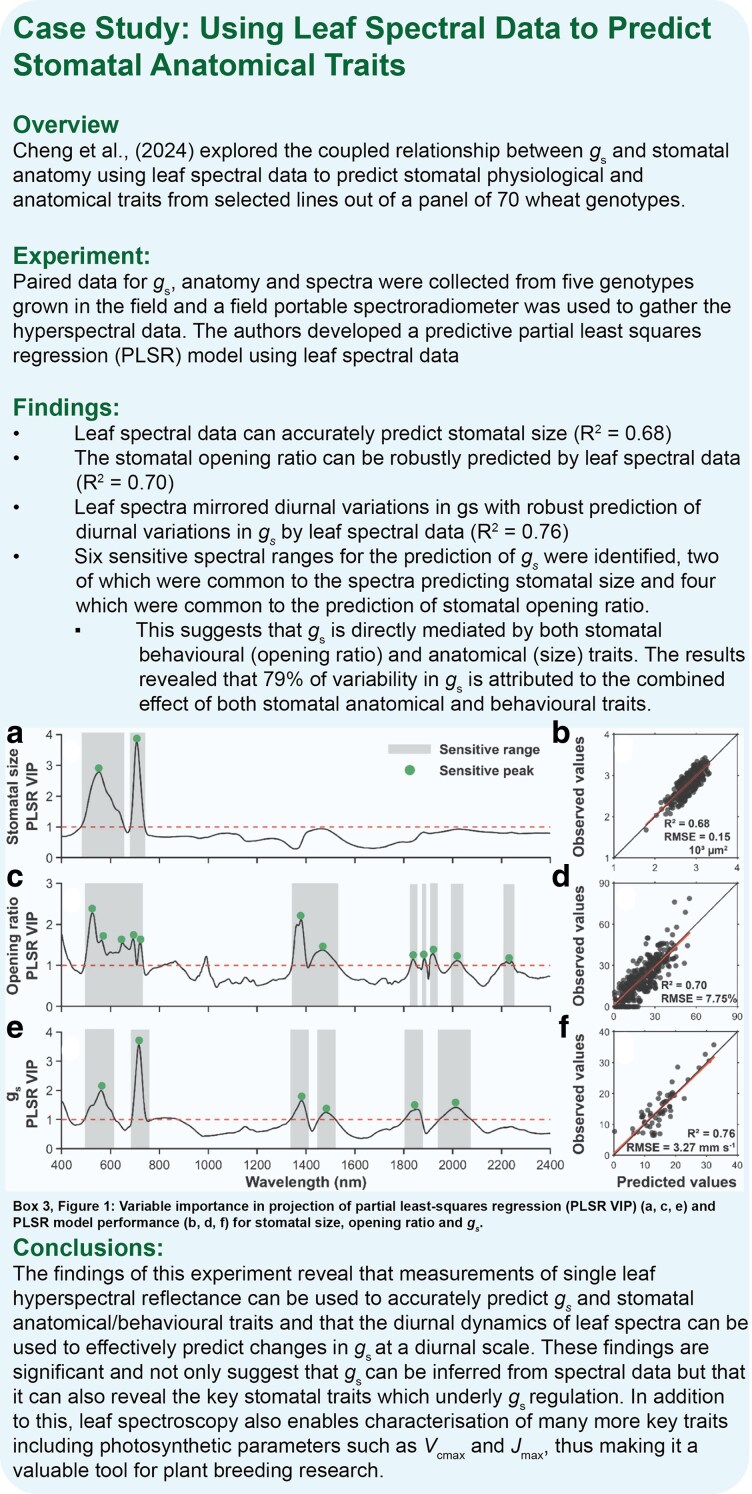

### Scaling up and scaling down

#### Remote sensing for scalability of stomatal assessment

The last decade has seen significant progress in the development of remote sensing techniques for plant phenotyping, including the use of unmanned aerial vehicles (UAVs) fitted with high resolution Red, Green, Blue (RGB), multispectral, HS, thermal, and light detection and ranging (LiDAR) sensors (for a comprehensive review of these sensors, see Gano *et al*. 2024). UAVs accurately capture variability and heterogeneity in field trials, operating quickly enough to minimize the impact of environmental fluctuations during data collection (Kim *et al.* 2021, Gano *et al.* 2024). Recent advances in remote sensing phenomics for crops like wheat, maize, and soybean highlight the need for larger-scale applications and standardized methodologies (Deery *et al.* 2019, Sobejano-Paz *et al.* 2020).

Thermal imaging holds significant potential for scalable stomatal trait assessment (Guilioni *et al.* 2008, Jones *et al.* 2018). Ground-based thermal imaging to accurately predict *g*_s_ has been demonstrated in wheat and other crops (Vialet-Chabrand and Lawson 2019, Banerjee *et al.* 2020, Ashfaq *et al.* 2022). While UAV-based thermal imaging for stomatal trait assessment at scale in wheat and maize has been carried out, it is difficult to implement (Brewer *et al.* 2022, Wang *et al.* 2024b). Limitations of microbolometers in contemporary thermal cameras (low spatial resolution, and low precision/accuracy) have prevented their deployment in plant breeding programmes (Grgić and Pušnik 2011, Wolf *et al.* 2016, Jiménez-Fortunato *et al.* 2025). On-ground calibration could overcome some of these limitations but is also difficult to practically implement. Canopy temperatures can also change rapidly with environmental fluctuations, posing a problem for imaging at scale. The lack of high precision sensors prevents capturing subtle differences in canopy temperature across large, closely related breeding populations (Kusnierek and Korsaeth 2014). For true adoption, next generation thermal imaging sensors featuring cooled microbolometers with minimal sensor drift are needed. These sensors must have high spatial resolution to capture detailed features and variability within plots, and high accuracy and precision to reliably detect differences in canopy temperature across populations of closely related germplasm (Yadav *et al.* 2022).

UAV HS imaging is heralded as the technology to bring physiological traits to the forefront of crop improvement (Liu *et al.* 2020a, Sarić *et al.* 2022). However, significant challenges exist in advancing from leaf scale using a spectroradiometer probe to flying a HS line scanner across a whole field at >20 m above the canopy. Unlike thermal cameras, highly capable HS imaging sensors deployable on UAVs exist, and on-ground spectral calibration is relatively easy, but translating data into meaningful physiological information remains challenging (Aasen *et al.* 2014, Matese *et al.* 2024). Ideally, models generated for predicting physiological traits at the leaf-scale could be used for HS data collected at a whole field-scale. However, the biggest challenges to achieving this are (i) HS imagers are passive, relying on the sun as a light source in contrast to the halogen lamp inside the spectroradiometer (Wang *et al.* 2017, Díaz *et al.* 2018); (ii) the canopy imaged at scale is more complex than a flat leaf surface imaged by a spectroradiometer; and (iii) adding spatial dimensions increases complexity and data volume (Atkinson and Aplin 2004, Liu *et al.* 2020b).

#### Scaling down with higher resolution for improved understanding

At an altogether different scale, groundbreaking advancements in 3D-microscopy using computer-based algorithms and AI modelling, have the potential to reshape our understanding of stomatal biology. These technologies unlock unprecedented insights into stomatal structures, such as pore geometry, spatial arrangement, and guard cell biomechanistic diversity including cell wall thickness, and architectural/physiological control mechanisms (Khoshravesh *et al.* 2022, Davaasuren *et al.* 2022). Computational modelling, integrated with 3D-imaging, provides valuable insights into how geometric differences in guard cells influence their biomechanical properties and the structural underpinnings of how plants adjust guard-cell cell wall mechanics to optimize stomatal responsiveness (Woolfenden *et al.* 2017, Yi *et al.* 2018). Guard-cell cell wall thickness, e.g. an often-overlooked trait, is pivotal to stomatal flexibility and dynamic closure efficiency. Studies suggest that thicker guard-cell cell walls can impede rapid stomatal closure during drought, potentially reducing WUE (Yi *et al.* 2019). By integrating these structural traits into functional-structural plant models, researchers can refine simulations of gas exchange and transpiration dynamics under fluctuating conditions. This level of detail supports the development of ‘stomatal efficiency indices’, which extend beyond traditional metrics like stomatal density and size, incorporating additional traits to provide a more holistic and comprehensive measure of stomatal performance.

Additionally, video microscopy and analysis offer exciting possibilities for new insights into stomatal behaviour. By capturing dynamic stomatal movements at high temporal resolutions, paired with gas exchange data, researchers could study how stomata respond to fluctuating environmental conditions in greater detail than ever before. This approach would allow observation of physical changes in stomatal aperture, providing a deeper understanding of the mechanisms driving stomatal responses to light, humidity, and CO₂ levels (Sun *et al.* 2021, Nicolaides *et al.* 2023). Integrating video microscopy with advanced image analysis algorithms could quantify the speed and extent of stomatal movements, revealing patterns that were previously undetectable, identifying key factors influencing stomatal efficiency and resilience.

#### Game changing data approaches

Fusing UAV-derived data layers with ground-truth measurements from porometry, gas exchange, microscopy and other ‘conventional’ techniques presents an opportunity to build robust predictive models of stomatal function and crop performance. Integrating modelling tools with physiological trait measurement bridges the gap between individual leaf-level trait measurement and field-level performance, enabling comprehensive, multiscale analyses of integrated stomatal physiology and anatomy traits. Multi-sensor fusion of HS imaging with thermal and LiDAR data would facilitate a multi-dimensional approach that could redefine phenotyping as we know it (Kusmec *et al.* 2021, Nguyen *et al.* 2023, Fei *et al.* 2023). Such platforms could simultaneously capture diverse data related to properties such as stomatal anatomy, conductance, leaf cooling patterns, and photosynthetic efficiency under stress, enhancing the accuracy of stomatal trait predictions. Furthermore, developing a standardized phenomics open access data platform adhering to FAIR data principles (findable, accessible, interoperable, and reusable) where researchers can share such datasets could further refine breeding efforts through a shared community of practice.

AI technologies are not just tools, they are catalysts for a paradigm shift in stomatal research. Generative AI models could predict long-term stress outcomes based on early anatomical and physiological measurements, as well as create synthetic datasets simulating stomatal behaviour under extreme conditions. These capabilities could help identify key traits linked to survival and resilience. AI algorithms could analyse vast datasets to identify optimal combinations of stomatal traits for specific environments, generating tailored, recommended breeding targets for stomatal traits. The power of AI lies in its ability to integrate diverse data streams—a comprehensive AI-driven platform would allow breeders to input environmental data (e.g. temperature, rainfall), desired traits (e.g. yield, drought resilience) and complex genetic profiles to identify wheat genotypes with maximum potential for improvement (Crossa *et al.* 2021, Xu *et al.* 2022).

Advances in ground-truthing measurement techniques, remote-sensing tools, deep learning, computer vision and data fusion approaches will facilitate a truly powerful toolkit for physiological trait assessment, enabling predictions of key traits across entire field trials using automated aerial- and ground-based platforms. This will allow physiological processes including stomatal function to be captured and monitored at much broader scales and smaller, more relevant timeframes. To unlock the full potential of these traits, we must embrace bold, transformative approaches.

### Rapid deployment of advantageous traits in appropriate germplasm

Leveraging multi-omics approaches is crucial for rapid deployment of advantageous stomatal traits in germplasm (Fig. 1). Integrating genomic, transcriptomic and metabolomic/proteomic data with phenomic data holds promise for uncovering the genetic and molecular mechanisms underlying stomatal behaviour and translating these to beneficial outcomes. Metabolomic profiling could reveal how stomatal responses influence metabolic pathways under stress (and vice versa), providing a more complete understanding of plant adaptation/acclimation mechanisms (Shi *et al.* 2020). Correlating high-throughput phenomics data with genomic and transcriptomic profiles could identify key genes and regulatory networks controlling complex traits including stomatal development and function (Chen *et al.* 2020, Su *et al.* 2021).

To effectively target these traits with genome-phenome analyses, genomic prediction and selection techniques are essential. These methods use dense genomic markers to unveil genetic insights into the traits of interest, identifying loci associated with desirable traits, pinpointing areas to target for breeding or editing, thus improving breeding efficiency and accuracy (Thakur *et al.* 2024). Genomic prediction and selection further enhance efficiency by using statistical models to predict the breeding value of individuals based on their genomic data, allowing faster and more precise selection of superior genotypes (Crossa *et al.* 2017).

Building on these insights, genome-wide association studies have already identified candidate genes for stomatal traits in wheat and other species (Bheemanahalli *et al.* 2021, Ferguson *et al.* 2021, Li *et al.* 2022b, Liu *et al.* 2025), laying important groundwork for targeted breeding and editing. New genome-editing tools such as CRISPR-Cas9 efficiently generate precise modifications within a single generation as opposed to 6–7 years via conventional techniques (Awan *et al.* 2022, Ahmad 2023, Zhou *et al.* 2023). For example, CRISPR has been used to knock out epidermal patterning factor (EPF) genes in rice, leading to increased stomatal density (Yin *et al.* 2017). While speeding up genetic modification does not guarantee translation into superior genotypes, combining editing with integrated multi-omics approaches enhances the precision of trait targeting. Such approaches offer promise for developing climate-resilient wheat (Rajotia *et al.* 2025, Abdallah *et al.* 2025). Modulation of EPFs such as *TaEPF1*, e.g. enhanced WUE and maintained yield under drought conditions by reducing stomatal density (Dunn *et al.* 2019). Similarly, the hexokinase *TaHXK3-2A* offers a promising target for regulating stomatal density and improving WUE in wheat (Li *et al.* 2022b). However, it is important to note that the adoption of these novel breeding technologies faces barriers, including stringent regulations and societal perceptions (Smyth 2020, Brookes and Smyth 2024). Overcoming these hurdles to translate such approaches from lab to field is vital for leveraging the full potential of gene editing in crop improvement (Karavolias *et al.* 2023).

The future of crop improvement lies in combining phenomic, genomic and transcriptomic approaches with cutting-edge modelling and AI which would help identify upstream regulators of stomatal development and function. Engineering stomatal regulatory networks, guided by AI-driven models, would facilitate the development of digital twins (virtual plant replicas) to simulate stomatal behaviour and its impact on crop performance. By combining real-time phenotyping data with advanced AI simulation, digital twins could predict how stomatal traits influence WUE, photosynthesis, and yield under climate stress, facilitating pre-emptive selection of genotypes with superior stomatal traits, thus reducing field trial costs and time (Verdouw *et al.* 2021, Pylianidis *et al.* 2021, Mitsanis *et al.* 2024). Additionally, digital twins could facilitate the design of ideotypes, theoretical plants with ideal trait combinations (Carbajal-Friedrich and Burgess 2024). Ideotypes would be transformative and would redefine the future of crop improvement: genotypes with precisely tailored stomatal characteristics, optimized for specific environments/stressors (Peladarinos *et al.* 2023).

Synthetic biology represents a transformative next-generation breeding approach (Goold *et al.* 2018) based on creating novel genetic pathways and cellular behaviours to enhance traits like stress tolerance, yield and nutritional quality (Tian *et al.* 2021, Wang *et al.* 2022, Zhang *et al.* 2023b). This could enable the precise engineering of stomatal formation, patterning, and responsiveness in ways that would not otherwise be possible, such as to improve synchrony between stomatal responses and mesophyll photosynthetic rates. While modelling suggests that synchronized responses of *A* and *g*_s_ could theoretically improve carbon assimilation by 20%–30%, the practical relevance of this in cereals, where stomata already respond relatively rapidly, remains debated, and steady-state *g*_s_ levels may be more influential for photosynthetic capacity in wheat (Lawson and Blatt 2014, Lawson *et al.* 2018). It could even be used to decouple stomata from carbon assimilation via a synthetic C4-pathway, as targeted by the recent C4 rice project (Furbank *et al.* 2023). Synthetic circuits could also be designed to dynamically regulate stomatal behaviour in response to environmental cues, optimizing WUE and photosynthetic performance under stress. Thinking outside the box, it could also be used to decouple plant water use and photosynthesis by engineering selective membranes which regulate the exchange of CO_2_ and H_2_O (Minguet 2015, da Fonseca-Pereira *et al.* 2022).

Advances in AI have further accelerated this field by improving the prediction of 3D protein structures and facilitating the design of *de novo* proteins with tailored functions. While synthetic biology has shown significant promise in crop breeding, its application to complex traits such as photosynthesis, carbon fixation, and metabolic pathways remains largely conceptual (Wurtzel *et al.* 2019). However, the rapid progress in related fields suggests that synthetic biology could soon become a cornerstone of plant breeding programmes. By integrating these tools, researchers could develop wheat varieties with ‘smarter stomata’ that respond even more rapidly to changing conditions to dynamically balance CO₂ uptake and water loss. Synthetic biology allows for more radical changes than conventional breeding or CRISPR would allow, enabling the creation of wheat varieties with vastly different or entirely novel stomatal patterning. This would facilitate a more holistic understanding of stomatal responses integrated with responses at a whole-plant level. Such innovations pave the way for a new era of precision breeding tailored to the challenges of a changing climate, enabling the plant ideotype to be reached sooner.

## Conclusion

The growing threat of heat and drought stress leaves no doubt: we must urgently develop new wheat varieties with enhanced stress tolerance. Wide phenotypic variation in wheat for stomatal traits highlights exploitable genetic variability. Although complex, by manipulating stomatal form and function, we can target crop improvement more effectively. An integrated approach that studies both stomatal physiology and anatomy is crucial to understand how anatomical traits influence physiological responses under stress. However, defining optimal stomatal trait ideotypes remains a major challenge, as different anatomical and physiological trait combinations may confer resilience depending on the stress type, intensity, and environmental context.

Emerging technologies are revolutionizing the collection and analysis of stomatal data at scales previously unimaginable, bridging the gap between small-scale leaf-level traits and large-scale field-level performance. Integrating these tools offers new avenues for breeding programmes to select genotypes with superior stomatal characteristics, unlocking the potential to develop high-yielding, climate-resilient wheat. However, achieving high-throughput stomatal phenotyping at field-scale requires further method development, particularly for UAV-based platforms. Approaches like multi-omics integration and digital twins hold the potential to deliver groundbreaking insights into stomatal traits. Next-generation breeding tools like CRISPR-Cas9 and synthetic biology offer unprecedented opportunities to develop ideotype plants with precisely engineered stomata that optimize CO_2_ assimilation and WUE, elevating the role of stomatal traits in breeding programmes, transforming them into pivotal targets for crop improvement.

However, novel tools are only part of the solution. A coordinated, collaborative effort among researchers is crucial to fully exploit their potential. With climate change accelerating, developing crop varieties that can withstand these challenges is increasingly urgent. These advancements bridge the gap between stomatal anatomy, physiology and breeding. Imagine a future where wheat varieties not only survive, but thrive under challenging environmental conditions, ensuring stable yields despite climate change. By improving carbon assimilation and WUE, these technologies and breakthroughs would reduce agriculture’s environmental footprint and secure food supplies. A concerted effort to harness these approaches could pave the way for a new era of crop science, addressing pressing challenges with innovative, science-driven solutions. Integrating these emerging technologies through collaborative research holds the promise of a transformative impact on society, food production and climate resilience, addressing the most pressing challenges of the current era.

## Data Availability

There is no data to provide associated with this paper.
